# Effect of Light Intensity on Morphology, Photosynthesis and Carbon Metabolism of Alfalfa (*Medicago sativa*) Seedlings

**DOI:** 10.3390/plants11131688

**Published:** 2022-06-25

**Authors:** Wei Tang, Haipeng Guo, Carol C. Baskin, Wangdan Xiong, Chao Yang, Zhenyi Li, Hui Song, Tingru Wang, Jianing Yin, Xueli Wu, Fuhong Miao, Shangzhi Zhong, Qibo Tao, Yiran Zhao, Juan Sun

**Affiliations:** 1 College of Grassland Science, Qingdao Agricultural University, Qingdao 266109, China; a20052123@126.com (W.T.); 20202203019@stu.qau.edu.cn (H.G.); xiongwd@qau.edu.cn (W.X.); yangchao@qau.edu.cn (C.Y.); lizhenyily@163.com (Z.L.); biosonghui@outlook.com (H.S.); xiaoxiang0203@126.com (T.W.); 20202103045@stu.qau.edu.cn (J.Y.); xueli0510@163.com (X.W.); miaofh@qau.edu.cn (F.M.); zhongsz@qau.edu.cn (S.Z.); taoqibo1992@163.com (Q.T.); zhaoyiran@qau.edu.cn (Y.Z.); 2 Department of Biology, University of Kentucky, Lexington, KY 40506-0225, USA; carol.baskin@uky.edu; 3 Department of Plant and Soil Science, University of Kentucky, Lexington, KY 40546-0312, USA

**Keywords:** alfalfa, light intensity, photosynthesis, growth, adaption

## Abstract

To understand how light intensity influences plant morphology and photosynthesis in the forage crop alfalfa (*Medicago sativa* L. cv. Zhongmu 1), we investigated changes in leaf angle orientation, chlorophyll fluorescence, parameters of photosynthesis and expression of genes related to enzymes involved in photosynthesis, the Calvin cycle and carbon metabolism in alfalfa seedlings exposed to five light intensities (100, 200, 300, 400 and 500 μmol m^−2^ s^−1^) under hydroponic conditions. Seedlings grown under low light intensities had significantly increased plant height, leaf hyponasty, specific leaf area, photosynthetic pigments, leaf nitrogen content and maximal PSII quantum yield, but the increased light-capturing capacity generated a carbon resource cost (e.g., decreased carbohydrates and biomass accumulation). Increased light intensity significantly improved leaf orientation toward the sun and upregulated the genes for Calvin cycle enzymes, thereby increasing photosynthetic capacity. Furthermore, high light (400 and 500 μmol m^−2^ s^−1^) significantly enhanced carbohydrate accumulation, accompanied by gene upregulation and increased activity of sucrose and starch-synthesis-related enzymes and those involved in carbon metabolism. Together, these results advance our understanding of morphological and physiological regulation in shade avoidance in alfalfa, which would guide the identification of suitable spatial planting patterns in the agricultural system.

## 1. Introduction

Light is one of the most important environmental factors influencing plant growth and development. Changes in light intensity, light quality and the photoperiod have impacts on plant morphology and metabolism [1]. Subsequently, plants can exhibit numerous adaptative strategies in response to the light environment [2]. When grown in the shade, many shade-intolerant plants (e.g., *Arabidopsis thaliana*) exhibit a well-known shade avoidance syndrome (SAS) that increases their adaptive and competitive ability [3]. The SAS is triggered by a reduction in light intensity perceived by photoreceptor cryptochromes, which in turn control adaptive responses [4]. These SAS responses range from development changes, such as increased leaf hyponasty, specific leaf area and ratio of palisade/spongy tissues; hypocotyl, petiole and stem elongation; reduced tillering (monocots)/branching (dicots); and increased internode length [5]. Physiological changes, such as decreased leaf carbon assimilation and enzyme activity, also occur [6]. The morphological changes in response to shading allow the plant to elongate and thereby gain access to unfiltered sunlight [7]. However, plant elongation due to shading comes at a cost. Plant carbon resources must be redirected to stems or petioles to promote their elongation at the expense of production of new leaves. Additionally, excessive stem elongation leads to plant lodging or mechanical injury, which decreases plant fitness [8]. In crop production, shading occurs for the low-tier plants, which decreases light intensity and changes the light quality to a low ratio of red light, especially in intercropping system, [9]. Subsequently, these plants respond to shade by inducing a series of adaptive morphological and physiological changes at the cost of assimilated resources, which eventually negatively affects yield [10]. Thus, gaining a better understanding of how crops adapt and respond to shade stress could help guide the design of crop cultivation in agriculture systems.

A range of light levels is a common approach for exploring how shading stress affects pigment accumulation and the photosynthetic capacity of leaves [1]. Light intensity can directly affect light harvesting by plants and lead to changes in the abundance of chlorophyll pigments and differences in the health status of PSII. Rascher et al. (2010) [11] found that low light led to higher levels of Chl a, b, an improved maximal PSII quantum yield (F_v_/F_m_) and an early onset of nonphotochemical quenching (NPQ), which increased light-capturing capacity. Similar results were obtained for seedlings of Chinese cabbage (*Brassica campestris*) [12] and sweet pepper (*Capsicum annuum*) [13]. These significant differences in photochemical efficiency can be viewed as adaptations to low light; therefore, their regulatory mechanisms have long been important areas of research.

Photosynthesis allows plants to convert light energy into chemical energy. The Calvin cycle is a series of biochemical redox reactions that take place in the stroma of chloroplasts, and they play a vital role in photosynthetic carbon fixation [14]. In shade-intolerant species, low photosynthesis due to low light reduces expression of genes and activity of the Calvin cycle enzymes involved in CO_2_ fixation and regeneration of rubisco-1, 5-bisphosphate (RuBP), thereby decreasing the potential for carbon assimilation in plants [15]. RuBP carboxylase or oxygenase (Rubisco) is the rate-limiting step of photosynthesis, and it catalyzes CO_2_ fixation in C_3_ plants [16]. Previously, it was reported that shade-associated with downregulation of the net photosynthetic rate was due to reduction in the amount or activity of Rubisco [17]. Photosynthesis is also catalyzed by other key enzymes, e.g., Rubisco activase (RCA) and fructose-1, 6-bisphosphatase (FBPase) [18]. Recent studies on soybean (*Glycine max*) and tomato (*Lycopersicon esculentum*) have shown that gene expression of the key enzymes involved in the Calvin cycle was downregulated in low but not high light [18,19]. However, the specific effects of light intensity on the photosynthesis processes in plants remain largely unknown. Therefore, levels of gene expression of the key enzymes of the Calvin cycle of plants grown at different light intensities need to be studied to elucidate the molecular mechanism of plant response to shading stress.

Alfalfa (*Medicago sativa* L.) is a high-quality forage for dairy cows and other livestock because of its high dry matter accumulation and high protein and soluble sugar content [20,21]. With increasing demand for food and the decreasing availability of arable land, grass/legume forage intercropping is gaining in popularity as a sustainable practice for low-input or resource-limited agricultural systems, such as maize–alfalfa and oat–alfalfa [22]. However, intercropped alfalfa plants often suffer from shade stress due to the reduced amount of intercepted sunlight. Subsequently, shading increases plants height and internodal distance, and reduces stems strength, which makes alfalfa plants susceptible to lodging, thereby reducing forage yield [23]. The SAS effects on alfalfa could be of high practical importance for intercropping systems, but the minimum amount of light required for alfalfa growth and development has received little research attention. To date, only one study has indicated that shade-intolerant alfalfa plants will delay flowering when grown in the shade (i.e., low ratio of red to far-red light) [24]. Research-based information is lacking on the effect of shading on the growth and physiological metabolism of *M. sativa* seedlings. Thus, it is important to investigate the adaptability of alfalfa responses to low light intensity, which would be useful information for determining proper plant spacing and strip configuration in intercropping systems.

The objective of our research was to determine how light intensity affects alfalfa seedling morphology and photosynthetic characteristics, as well as the key enzymes involved in the Calvin cycle and carbon metabolism coupled with expression of these genes. Here, alfalfa seedlings were exposed to five levels of light intensity for 14 days in a climate room, and their morphological and physiological responses were investigated. We hypothesized that a brief exposure to low light would increase leaf hyponasty and stem elongation but downregulate expression of genes for the key enzymes involved in the Calvin cycle and carbon metabolism, resulting in a synergistic decrease in photosynthetic rates and accumulation of dry matter.

## 2. Results

### 2.1. Morphological Characteristics

**Figure 1:**
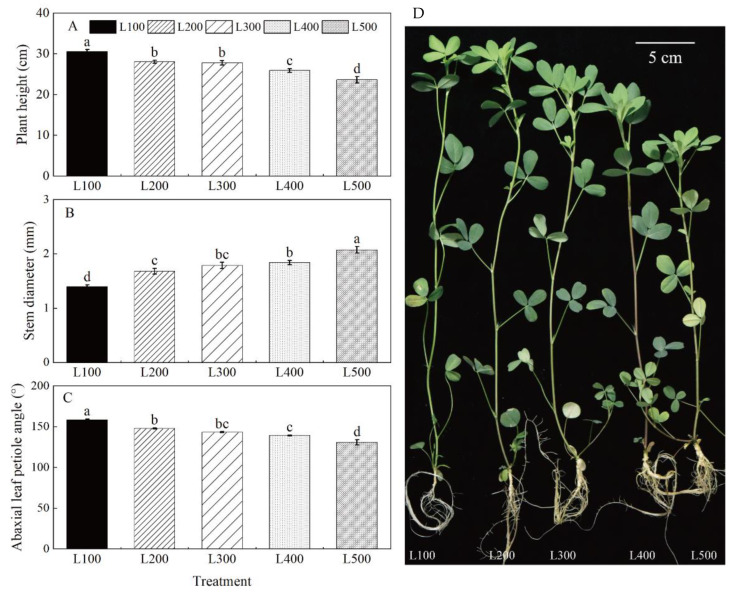
Changes in phenotype and plant traits of alfalfa as affected by light treatments. The plant height (**A**), stem diameter (**B**), abaxial leaf petiole angle (**C**) and plant phenotype (**D**) of alfalfa plants under different light intensity treatments. L100, L200, L300, L400 and L500 refer 100, 200, 300, 400 and 500 µmol m^−2^ s^−1^, respectively. Vertical bars indicate 1 s.e. of the mean (*n* = 4). Different lowercase letters on the different bar mean significant differences (*p* < 0.05).

Light treatment had a significant effect on alfalfa morphological characteristics (i.e., plant height, specific leaf area, abaxial leaf petiole angle and stem diameter) (*p* < 0.001) (Figure 1, Table 1 and Table S2). Maximum plant height, specific leaf area and abaxial leaf petiole angle were measured in L100; these are the traits that decreased with increased light intensity. However, the highest and lowest stem diameters were measured for plants at L500 and L100, respectively. In addition, shoot dry matter (SDM), root dry matter (RDM) and the root-to-shoot ratio (RSR) were significantly affected by light treatments (*p* < 0.001) (Table S3). The SDM, RDM and RSR of alfalfa plants in L500 were significantly higher than those in L100. For the most part, the RSR did not differ significantly between L300, L400 and L500 (Table 1).

Root morphology parameters, including root length (RL), surface area (RSA), volume (RV) and diameter (RD), varied among light treatments (Table S3). These parameters increased with increasing light up to L500 compared to the L100 treatment (Table 2). Increased light significantly increased RL by 22.8 to 182.5%, RSA by 26.0 to 353.2%, RV by 42.2 to 925.9% and RD by 4.3 to 84.5%. RD did not differ significantly from L300 to L500.

### 2.2. Leaf Pigment and Nitrogen Content

Chlorophyll a (Chl a), Chlorophyll b (Chl b), carotenoids (Car), Chl a + b, Chl a/b and leaf nitrogen content (LN) were significantly affected by light treatment (Table S4). Increased light intensity from L100 to L500 decreased Chl a, Chl b, Chl a + b and Car contents, while Chl a/b increased (Table 3). Chl a, Chl b, Chl a + b and Car contents for plants in the L500 treatment were decreased by 27.8%, 49.5%, 32.9% and 25.9% (*p* < 0.01), respectively, compared to L100, but those for plants grown at L400 and L500 did not differ significantly. Chl a/b was 17.5% (*p* < 0.01) higher in L500 than in L100. In addition, increased light intensity decreased LN content, and at L500, LN content decreased by 50.6% (*p* < 0.001) compared to L100.

### 2.3. Photosynthetic and Chlorophyll Fluorescence Characteristics

**Figure 2:**
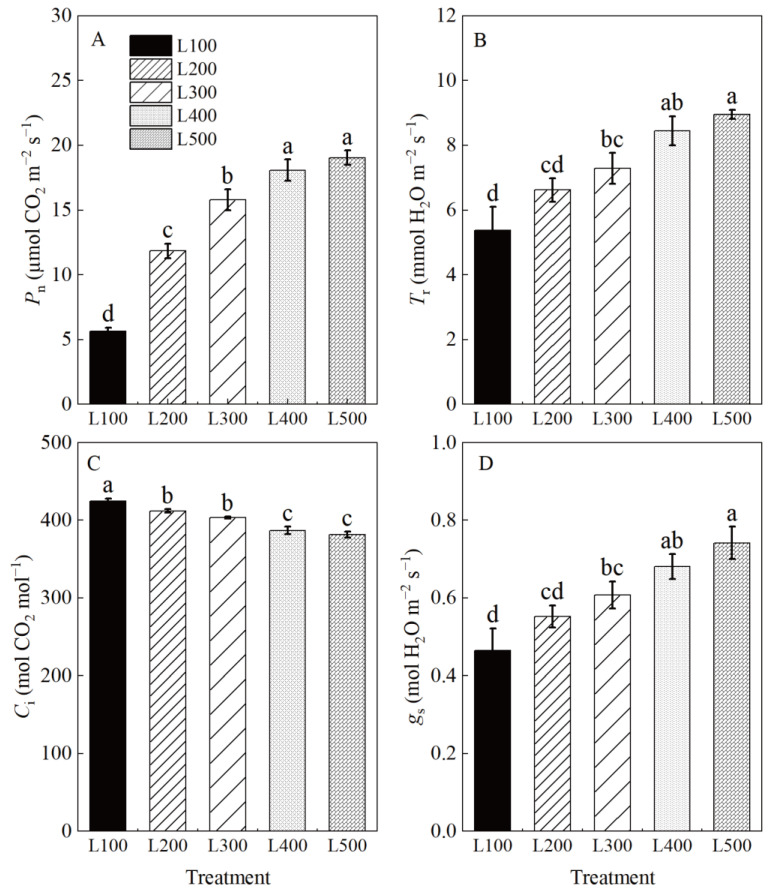
Photosynthetic characteristics of alfalfa leaves under different light treatments. L100, L200, L300, L400 and L500 refer 100, 200, 300, 400 and 500 µmol m^−2^ s^−1^, respectively. Net photosynthetic rate (*P*_n_) (**A**), transpiration rate (*T*_r_) (**B**), intercellular CO_2_ concentration (*C*_i_) (**C**), stomatal conductance and (*g*_s_) (**D**). Vertical bars indicate 1 s.e. of the mean (*n* = 4). Different lowercase letters on the different bar mean significant differences (*p* < 0.05).

The photosynthetic characteristics of alfalfa plants varied among light treatments (Table S5, Figure 2). The maximum net photosynthetic rate (*P*_n_), transpiration rate (*T*_r_) and stomatal conductance (*g*_s_) values of alfalfa plants were at L400 and L500, whereas intercellular CO_2_ concentration (*C*_i_) was highest at L100 to L300. On average, the net photosynthetic rates, *T*_r_ and *g*_s_ of alfalfa plants, were significantly increased by 230, 62 and 52%, respectively, (*p* < 0.01) at L400 and L500 compared to L100. However, intercellular CO_2_ concentration at L400 and L500 decreased by 8.9 and 10.1% (*p* < 0.001), respectively, compared to L100. The photosynthetic characteristics of alfalfa leaves did not differ significantly at L400 and L500. The increased *P*_n_ at L400 and L500 suggests that high light intensity was positively related to increased *g*_s_ and *T*_r_, but negatively related to decreased *C*_i_ in alfalfa plants.

**Figure 3:**
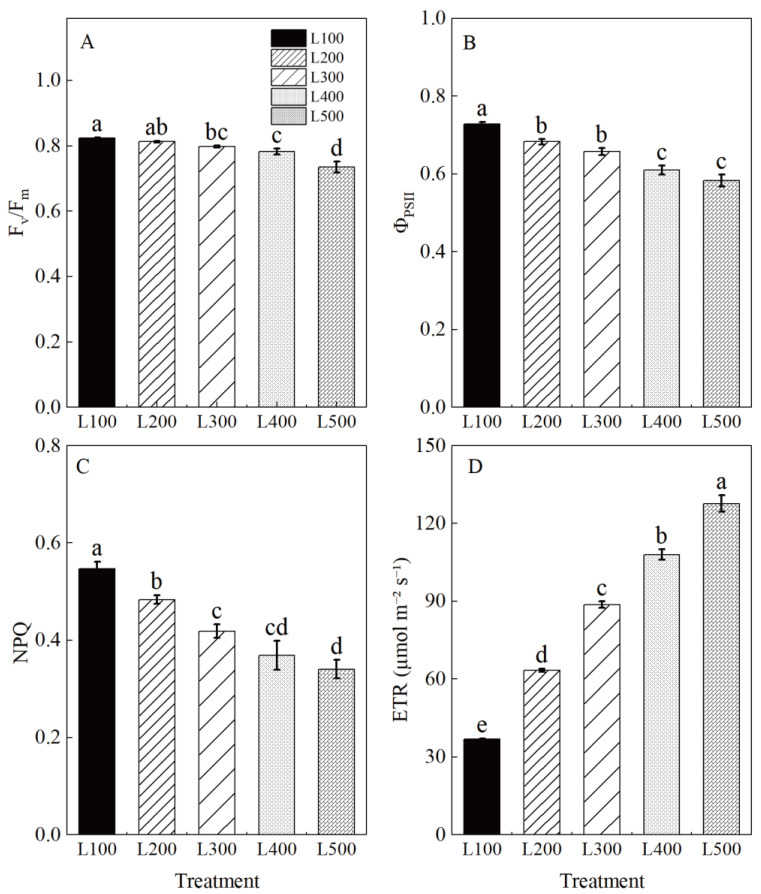
Chlorophyll fluorescence characteristics of alfalfa leaves under different light treatments. L100, L200, L300, L400 and L500 refer 100, 200, 300, 400 and 500 µmol m^−2^ s^−1^, respectively. Maximal PSII quantum yield (Fv/Fm) (**A**), effective PSII quantum yield (Φ_PSII_) (**B**), non-photochemical quenching (NPQ) (**C**) and electron transport rate (ETR) (**D**). Vertical bars indicate 1 s.e. of the mean (*n* = 4). Different lowercase letters on the different bar mean significant differences (*p* < 0.05).

Chlorophyll fluorescence characteristics, including maximal PSII quantum yield (F_v_/F_m_), effective PSII quantum yield (Φ_PSII_), non-photochemical quenching (NPQ) and the electron transport rate (ETR), were significantly affected by treatment (Table S6, Figure 3). Figure 3 shows the difference in absorbed radiation energy of alfalfa leaves in response to light treatments. The F_v_/F_m_, Φ_PSII_ and NPQ of alfalfa plants grown in the low-light treatments were significantly higher than those in the high-light treatments. Furthermore, L100 increased the F_v_/F_m_, Φ_PSII_, and NPQ by 12.0, 24.9 and 60.8%, respectively, but it decreased the ETR by 71.2% (*p* < 0.001) compared to L500. These results indicate that the original activity of the PSII reaction center was increased, and the transformation efficiency of primary light energy was improved in the low-light-intensity adaption of alfalfa.

### 2.4. Leaf Non-Structural Carbohydrate Contents

**Figure 4:**
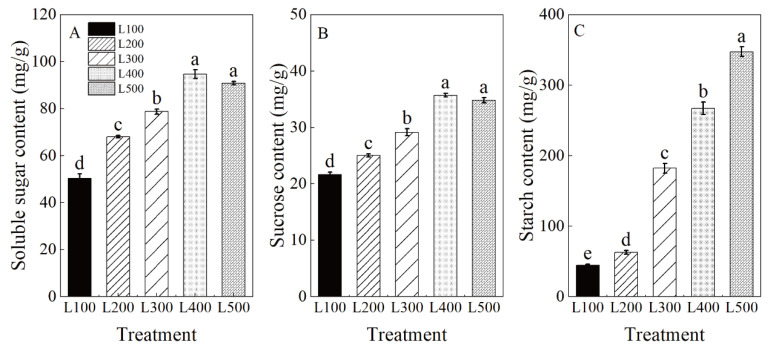
Changes in carbon balance of alfalfa plants under different light treatments. L100, L200, L300, L400 and L500 refer 100, 200, 300, 400 and 500 µmol m^−2^ s^−1^, respectively. Soluble sugar content (**A**), sucrose content (**B**) and starch content (**C**). Vertical bars indicate 1 s.e. of the mean (*n* = 4). Different lowercase letters on the different bar mean significant differences (*p* < 0.05).

Soluble sugar (SS), sucrose and starch (St) were significantly affected by light treatments (*p* < 0.001) (Table S7). As expected, the content of SS, sucrose and St in leaves increased significantly with increased light intensity (Figure 4). The highest SS, sucrose and St contents in leaves were measured in the high-light treatments (i.e., L400 and L500) compared to low-light treatments (i.e., L100 and L200).

### 2.5. Gene Expression and Enzymatic Activity

**Figure 5:**
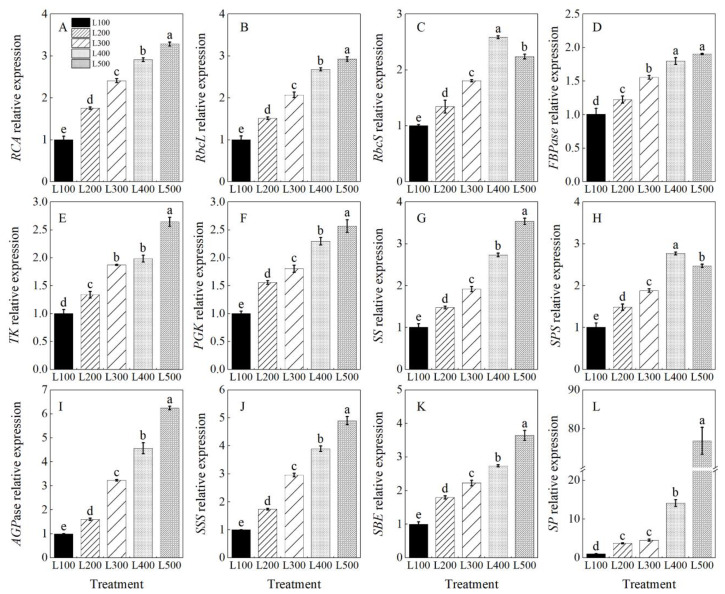
Changes in level of gene expression of alfalfa plants growing in different light treatments. L100, L200, L300, L400 and L500 refer 100, 200, 300, 400 and 500 µmol m^−2^ s^−1^, respectively. Rubisco activase (*RCA*, (**A**)), Rubisco large subunit (*RbcL*, (**B**)), Rubisco small subunit (*RbcS*, (**C**)), Fructose-1,6-bisphosphatase (*FBPase*, (**D**)), Transketolase (*TK*, (**E**)), Phosphoglycerate kinase (*PGK*, (**F**)), sucrose synthase (*SS*, (**G**)), sucrose phosphate synthase (*SPS*, (**H**)), ADP-glucose pyrophosphorylase (*AGPase*, (**I**)), soluble starch synthase (*SSS*, (**J**)), starch-branching enzyme (*SBE*, (**K**)) and starch phosphorylase (*SP*, (**L**)). Vertical bars indicate 1 s.e. of the mean (*n* = 3). Different lowercase letters on the different bar mean significant differences (*p* < 0.05).

The expression levels of genes encoding sucrose synthase (*SS*), sucrose phosphate synthase (*SPS*), starch synthase (*AGPase*, *SSS*, *SBE* and *SP*) and those involved in the Calvin cycle (such as *RCA*, *RbcL*, *RbcS*, *FBPase*, *TK* and *PGK*) were quantitatively analyzed, and they were significantly affected by the light treatments (Table S8). The relative expression levels of these genes were upregulated with increasing light intensity up to L500 compared to the L100 treatment (Figure 5A–L). In addition, the relative expression of *RbcS* in the L400 treatment was 2.6 (*p* < 0.001) times higher than that in the L100 treatment.

**Figure 6:**
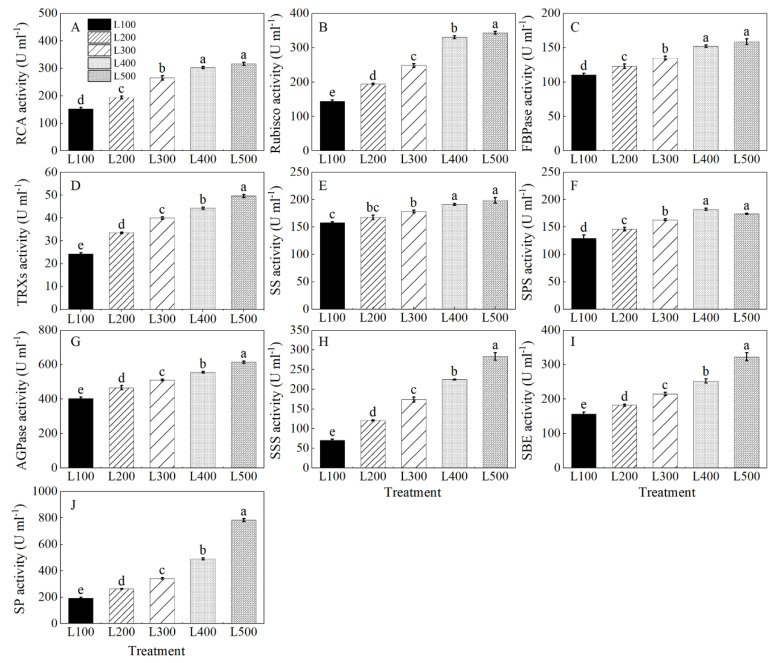
Changes in enzymatic activity of alfalfa plants growing in different light treatments. L100, L200, L300, L400 and L500 refer 100, 200, 300, 400 and 500 µmol m^−2^ s^−1^, respectively. Ribulose-1,5-bisphosphate carboxylase/oxygenase activase (RCA, (**A**)), ribulose-1,5-bisphosphate carboxylase/oxygenase (Rubisco, (**B**)), fructose-1, 6-bisphosphatase (FBPase, (**C**)), thioredoxin reductase (TRXs, (**D**)), sucrose synthase (SS, (**E**)), sucrose phosphate synthase (SPS, (**F**)), ADP-glucose pyrophosphorylase (AGPase, (**G**)), soluble starch synthase (SSS, (**H**)), starch-branching enzyme (SBE, (**I**)) and starch phosphorylase (SP, (**J**)), Vertical bars indicate 1 s.e. of the mean (*n* = 4). Different lowercase letters on the different bar mean significant differences (*p* < 0.05).

The activity of ribulose-1,5-bisphosphate carboxylase/oxygenase activase (RCA), Rubisco, fructose-1, 6-bisphosphatase (FBPase), thioredoxin reductase (TRXs), sucrose synthase (SS), sucrose phosphate synthase (SPS), adenosine diphosphate glucose pyro-phosphorylase (AGPase), soluble starch synthase (SSS), starch-branching enzyme (SBE) and starch phosphorylase (SP) of alfalfa plants varied with the light treatment (Table S9). Rubisco, RCA, FBPase, TRXs, SS, SPS, AGPase, SSS, SBE and SP activities of alfalfa plants increased gradually with increasing light intensity from L100 to L500, and the highest values were at L400 and L500 (Figure 6A–J). On average, the activities of RCA, Rubisco, FBPase, TRXs, SS, AGPase, SSS, SBE and SP were higher (*p* < 0.001) at L500 than at L100. In addition, SPS activity of alfalfa plants was the highest at L400, which was 40.8% higher than at L100 (*p* < 0.001).

## 3. Discussion

The shade avoidance syndrome is an adaptive response that increases fitness in a shaded environment by reshaping the plant morphology and modifying physiological processes [25]. Our study examined the morphology, photosynthesis, carbohydrate metabolism and the expression of genes related to photosynthesis and carbon metabolism in leaves of alfalfa seedlings grown under low-to-high light intensities. Alfalfa seedlings displayed a degree of morphological adaptation, photosynthetic tolerance and carbon balance to the light intensity attenuation. Nevertheless, excessively low light significantly accelerated stem elongation and inhibited the photosynthetic process (e.g., net photosynthetic rate and Rubisco activity) of the seedlings. Low light intensity also negatively impacted production of photoassimilates (e.g., soluble sugar and starch), which in turn restricted growth and dry matter accumulation. Therefore, our results reveal the effects of simulated shade on phenotypic, physiological and expressional regulation in alfalfa, and thus provide insight into shade regulation in intercropping systems. The implications of these results are considered below.

### 3.1. Light Intensity Affects Morphological Characteristics

When the shading level is increased, plants adjust through a series of growth responses, such as increasing plant height, leaf hyponasty, leaf area and specific leaf area [5,26]. In our study, plant height in the low-light treatments was significantly higher than that in the high-light treatments, whereas the reverse occurred with stem diameter. When plants are shaded, more carbohydrates are used to increase stem length than to increase stem diameter. Increased plant height may result in an increase in the amount of light received by the leaves [27]. In agriculture production, shading increases plant height and reduces stem diameter and eventually increases lodging, which hinders the transportation of nutrients, water and photosynthetic products and causes huge yield losses [28]. Additionally, light intensity affects leaf position and expansion, which play important roles in the process of irradiation interception and photosynthesis [19]. There was a greater increase in leaf hyponasty and specific leaf area of alfalfa leaves in the low-light (100–300 μmol m^−2^ s^−1^) than in high-light (400–500 μmol m^−2^ s^−1^) treatments, which increased light interception by the leaves. This finding is in agreement with Song et al. (2015) [29], who found that increased leaf area and leaf angle could optimize the absorbed light for carbon fixation, which in turn increased photosynthetic capacity, thereby counteracting the stress of growing in low light. Additionally, the abaxial leaf petiole angle and specific leaf area (SLA) can increase under low photosynthetic photon flux density (PPFD) compared to high-PPFD interception conditions [5]. Thus, plants grown under high light have a decreased SLA, which in turn mitigates or prevents leaf internal structure damage caused by excessive light intensity. Therefore, morphological changes in resource-harvesting organs can contribute to increased photosynthetic efficiency, which helps override light limitation stress.

Similarly, we found that increased light intensity significantly changed the morphology of alfalfa seedlings by increasing dry matter accumulation in the shoot and root, resulting in more robust seedlings. We also confirmed results from a previous study by Pan et al. (2020) [18], which showed that increased light intensity significantly increases dry matter accumulation of each organ, indicating that in turn additional photosynthates are partitioned among all organs. It is possible that increased leaf growth (source) drives root growth (sink) and thus increases the ability of plants to acquire more water and nutrients, which could be an optimal way to maintain a source–sink balance under high-light conditions [30]. Our results also are in agreement with previous research on peanut (*Arachis pintoi*) [31], suggesting that plants allocate more resources to the part that is acquiring the resource that is currently the most limiting [32]. In addition, the morphological differences in alfalfa seedlings undergoing different light treatments may be due to alterations in the molecular regulation networks or endogenous plant hormones [33,34], which deserve further investigation.

### 3.2. Effect of Light Intensity on Chlorophyll Content and Chlorophyll Fluorescence Characteristics

The chlorophyll content of leaves is an important part of the light-harvesting system, and it is affected by shading [35]. According to our study, significant changes were observed in Chla, Chl b, Chl a + b and Car contents, which increased in low light. The chlorophyll content of leaves of maize (*Zea mays*) plants grown in low light intensity was significantly higher than that of leaves grown in high light [36], which agrees with our results. Increased Chl b content could be a typical response to low-light conditions that allows shade-intolerant plants to capture more photosynthetically efficient blue light, thereby stimulating adaptive photomorphogenesis and alleviating the negative impacts of shade stress on photosynthetic activities [37]. Yi et al. (2020) [38] also found that adequate CO_2_ assimilation and fixation promoted sugar accumulation and decreased pigment–protein complexes in leaves in high light intensity, resulting in senescence and chlorophyll degradation. Furthermore, decreased chlorophyll content could prevent excess light from damaging the photosynthetic metabolic process, which would enhance plant fitness under high-light conditions [39].

Chlorophyll fluorescence parameters can reflect the photosynthetic regulation ability of plants, and the efficiency of photochemistry can be used to evaluate the physiological responses of plants to environment stress [11]. Fv/Fm is quantum photochemical yield, and it is the ratio of number of quanta transferred to the QA acceptor to number of quanta absorbed by PSII. The high Fv/Fm value observed in L100-treated alfalfa seedlings indicates that resistance to photoinhibition was improved. Sun et al. (2014) [17] reported that Fv/Fm also increased significantly with light intensity attenuation in cucumber (*Cucumis sativus*) leaves, which displayed a decreased degree of photoinhibition and an increase in the openness and electron transport efficiency of PSII. In addition, the efficiency of PSII photochemistry (ΦPSII) can be used to reveal the physiological state of plants, and non-photochemical quenching (NPQ) is linearly related to excited energy dissipation of plants [40]. In our study, increased ΦPSII was accompanied by a corresponding increase in NPQ in the leaves grown under low light. It is possible that heat dissipation increases enough to protect the PSII photosystem from photoinhibition in the leaves grown in a low-light environment [38]. Our results suggest that the original activity of the PSII reaction center was increased, and the transformation efficiency of primary light energy was improved in the low-light adaption of alfalfa. However, the ETR was significantly higher in L400 and L500, further indicating that increased light intensity could enhance the electron transport from PSII to PSI. Similar results were also found in soybean under optimum light conditions (400 and 500 μmol m^−2^ s^−1^) in a growth chamber [19].

### 3.3. Effect of Light Intensity on Photosynthetic Characteristics and Carbohydrate Accumulation

The net photosynthetic rate (*P*_n_), transpiration rate (*T*_r_) and stomatal conductance (*g*_s_) gradually increased with an increase in light intensity, whereas the reverse occurred in the intercellular CO_2_ concentration (*C*_i_) of alfalfa plants. The main factors influencing *P*_n_ were *g*_s_ and *C*_i_, both of which are indispensable for determining the primary cause of change in *P*_n_ [36,41]. These results suggest that increased *P*_n_ under high light intensity could be due to increased stomatal opening, which would increase net CO_2_ assimilation and water vapor exchange, thus promoting photosynthesis [42]. Transpiration acts as a driving force behind the absorption and transportation of water and inorganic irons to the above-ground part of the plant [43]. Additionally, loss of water through the stomata is an important heat dissipation mechanism [37]. We found that under optimum light conditions (L400 and L500), the *P*_n_, *T*_r_ and *g*_s_ of alfalfa can be increased and the *C*_i_ reduced, which in turn enhanced photosynthesis in alfalfa plants.

As in previous studies [44], we found that sucrose, starch and total soluble sugar contents in alfalfa leaves were significantly improved with increased light intensity. Our results indicate that increased light (L400 and L500) increased specific leaf weight and the net leaf-level photosynthetic rate, which improved the number of photosynthates stored in the leaves. However, low light intensity (L100) could cause carbohydrate loss due to inhibition of photosynthesis and inhibit plant growth. Low light intensity decreased electron transfer and net photosynthetic rates, thereby exerting a negative impact on accumulation of photosynthetic products by the seedlings [19]. Furthermore, carbohydrates also serve as carbon reserves (e.g., sucrose and starch) and are stored in plant organs. Sucrose is one of the main sources of carbon and energy in plants. In our study, sucrose content was significantly higher under high light than low light, suggesting that plants grown in high light possessed stronger photosynthesizing leaves (source tissues) that in turn increased the sucrose produced by photosynthesis for supplying the demand of growing tissues [1]. In addition, starch reserves provide an immediate available energy source that may act as a buffer when environmental conditions are not optimal for photosynthesis (e.g., shade and cloudy days) [45,46]. Less carbon was partitioned to starch synthesis at low light intensity than at high light intensity [47,48]. Our results agree with those of Dayer et al. [45] and Jian et al. (2019) [49], who found that the assimilate demand of plants exceeds the photosynthetic rate under shaded conditions, suggesting that degradation of starch reserves into soluble sugars could be used to support metabolism during a period of moderate shading stress [50].

### 3.4. Effect of Light Intensity on Enzymatic Activity

In C3 plants, photosynthesis is mostly regulated by the activity of Calvin cycle enzymes, including RCA, Rubisco, FBPase and TRXs, which are recognized as very early and fast responses of plants to shading stress [51,52]. Our results show that Rubisco activity in high-light-treated alfalfa plants was significantly higher than that in plants grown in low-light conditions. Similar results were reported by Feng et al. [19], suggesting that the activity of Rubisco increases with increasing light intensity, which could increase carbon assimilation and RuBP regeneration in the Calvin cycle [17]. We also found that decreased *P*_n_ in alfalfa grown under low light intensity was accompanied by reductions in RAC and Rubisco activity and the transcriptional levels of most genes (*RCA*, *RbcL*, *RbcS*, *FBPase*, *TK* and *PGK*) involved in the Calvin cycle. Our results are in accordance with those of Zhang et al. (2020) [53], suggesting that restriction of CO_2_ carboxylation in the Calvin cycle is a result of impaired activity of RCA. The RCA could remove inhibitors bound to Rubisco, and thus a decline in the activity of RCA indirectly causes the decreased CO_2_ assimilation rate in low-light-grown seedlings [54]. Further, the activation state of RCA, which is controlled by the redox state of the cell, is sensitive to light intensity, and the proper regulation of RCA activity is also vital for acclimation to light fluctuation in *Arabidopsis* [55,56]. Therefore, depression of photosynthetic capacity induced by low light could be attributed to deceleration of the Calvin cycle [53].

Light intensity also plays a vital role in regulating the enzymes related to sucrose and starch biosynthesis [57,58]. The relative expression levels of *SPS*, *SS*, *AGPase*, *SSS*, *SBE* and *SP* were enhanced, and their encoding enzymes showed higher activities in the high-light treatments than low-light treatments, resulting in improved production of sucrose and starch [19]. Similar results also are reported for *Arabidopsis* [59] and soybean [19], suggesting that changes in light intensity equally promote the activities of SS, SPS and SSS and increase the sucrose and starch content, which improve plant growth and development. Therefore, the enzymatic activities of sucrose synthesis and starch synthesis enzymes play a vital role in regulating carbohydrate production, which is important in controlling storage of carbon reserves and growth of cells and tissues in plants under low light [60,61]. Therefore, the enzymatic activities for increasing sucrose and starch contents in alfalfa plants were the most effective in the L400 and L500 treatments.

## 4. Conclusions

In the present study, we studied the impacts of low and high levels of light on the morphology, photosynthesis characteristics and carbon metabolism of alfalfa seedlings and found that they are sensitive to shade. Increased light intensity (400 to 500 μmol m^−2^ s^−1^) enhanced the growth and dry matter accumulation, photosynthesis, carbon assimilates (sucrose and starch) and leaf enzymatic activities of enzymes related to the Calvin cycle by upregulating the important corresponding synthase genes, which positively improved carbon balance. In addition, alfalfa seedlings displayed a shade avoidance syndrome that increased their adaptive ability to compensate for low-light limitation (L100) but at the expense of dry matter and carbohydrate accumulation. The results allow us to understand the morphology, physiology and molecular behavior of plants exposed to different light intensities. Thus, gaining a more complete mechanistic picture of how alfalfa plants adapt and respond to light levels would provide useful support for guiding spatial arrangement of the alfalfa canopy in an intercropping system, thereby improving food production and ensuring higher yields.

## 5. Materials and Methods

### 5.1. Plant Material and Growth Conditions

The experiment was conducted in LED climate rooms located in the basement of the College of Grassland Science, Qingdao Agricultural University, Qingdao, China. Light intensity and spectral irradiance (λ = 350–800 nm) were measured by HR550 (Hipoint Inc., Gaoxiong, Taiwan), and the spectral distributions are shown in Figure S1. The photoperiod was 12 h with a 25 °C day temperature, 20 °C night temperature and a relative humidity of 60%.

*M. sativa* L. cv. Zhongmu 1 was chosen for the studies on phenotypic responses to growth conditions. Before the experiment, alfalfa seeds were surface-sterilized by 75% ethanol for 1 min and rinsed with deionized water for 5 min and germinated on wet sterile Whatman No. 1 filter paper in a daily 8 h light from white fluorescent tubes (Sanpai Corporation, Shanghai, China) with a mean photon flux density of 60 μmol m^−2^ s^−1^ (400–700 nm) at 25 °C. After seed coat rupture and cotyledon expansion at 5 days, 10 uniform-sized sprouting alfalfa seedlings were transplanted into a separate plugged hole in a foam sheet floating in a 3.3 L plastic container filled with half-strength Hoagland’s solution. These containers were placed in the LED climate room with a light intensity of 500 μmol m^−2^ s^−1^. When the first trifoliate leaf was well-developed, the pre-cultured seedlings were transferred to five light treatments. Photosynthetic photon flux density (PPFD) was 100 μmol m^−2^ s^−1^ (L100), 200 μmol m^−2^ s^−1^ (L200), 300 μmol m^−2^ s^−1^ (L300), 400 μmol m^−2^ s^−1^ (L400) and 500 μmol m^−2^ s^−1^ (L500), and light quality was the same in all treatments. The highest light intensity was chosen as it was used to grow alfalfa seedlings under laboratory conditions, and the lowest light intensity can be considered comparable to the natural shade under a closed oat forage canopy under clear-sky conditions (82–116 μmol m^−2^ s^−1^, unpublished analysis of solar radiation penetrating closed canopy in the alfalfa–oat intercropping system by W. Tang). Four containers were placed in each light treatment, and each container had 10 seedlings. The plastic containers were moved daily to avoid boundary effects, and Hoagland’s solution was renewed every 3 days and kept aerated by air-spraying. Every treatment was performed with four replicates.

### 5.2. Plant Morphology Parameters

After 14 d of treatment, four plants from each treatment were randomly selected, and the main growth parameters were measured: plant height, stem diameter, abaxial leaf petiole angle and leaf area. Leaf area was determined using an Li-3000 leaf area meter (Li-Cor Inc.). Specific leaf area (SLA) was calculated as: SLW = leaf area / leaf dry weight. Root morphology parameters were digitized with a LA-S scanner and analyzed using WinRhizo software (LA-S, Wanseng, China). After this, shoot and root samples were heated at 105 °C for 30 min and then dried to a constant weight in a fan oven at 75 °C. Dry matter was expressed as mg plant^–1^. Root-to-shoot ratio was also calculated.

### 5.3. Photosynthetic Pigment Content and Leaf Nitrogen Content

After 14 d of treatment, the third trifoliate leaf on alfalfa seedlings was collected for photosynthetic pigment and leaf nitrogen content measurement. Chlorophyll a (Chl a), Chlorophyll b (Chl b) and carotenoids (Car) were extracted from all leaf samples. Six leaf discs (0.6 cm in diameter) were cut from the middle part of each middle leaflet with a puncher, and they were placed in 25 mL of 95% acetone in the dark for 24 h, at which time the sample was colorless. Concentration of Chl a, Chl b and Car was measured at wavelengths of A_663_, A_645_ and A_470_ nm, respectively, using a UV spectrophotometer (UV-2700, Shimadzu, Kyoto, Japan), and calculated according to Pan et al. (2020) [18].

The dried leaf tissues were ground to obtain homogenous samples. A subsample of about 0.1 g was then digested with 5 mL of concentrated H_2_SO_4_ for 2 h in 420 °C, and K_2_SO_4_ and CuSO_4_·5H_2_O (K_2_SO_4_:CuSO_4_·5H_2_O = 10:1) as the catalyzer. Finally, the leaf nitrogen concentration was measured by an automatic flow injection analyzer (AA3, SEAL).

### 5.4. Photosynthesis Parameters

After 14 d of treatment, Li-6800 portable photosynthesis system (LI-COR Inc., Lincoln, NE, USA) was used for photosynthetic parameter measurement on the third fully expanded leaf of alfalfa seedlings. All parameters, including net photosynthetic rate (*P*_n_), transpiration rate (*T*_r_), intercellular CO_2_ concentration (*C*_i_) and stomatal conductance (*g*_s_), were measured under steady photosynthetic photon flux density in the leaf chamber, which was set to the same level as the relevant treatments. An Li-COR standard red–blue chamber set at 25 °C and a CO_2_ concentration of 460 µmol mol^−1^ were used.

### 5.5. Chlorophyll Fluorescence Measurements

After measuring the rate of photosynthesis, a Chlorophyll a fluorescence measurement was performed with the third fully expanded leaf on the alfalfa seedlings. Before measurement, each seedling was held in a dark chamber for 30 min prior to being submitted to the chlorophyll fluorescence procedure using an Li-6800 portable photosynthesis system. Fluorescence parameters characterizing the state of the photosynthetic apparatus were calculated on the basis of induction fluorescence curves obtained using data from the JIP test, which is usually used to evaluate the state of PSII. On the basis of induction fluorescence curves (OJIP curves), the following parameters, which characterize the maximal PSII quantum yield (F_v_/F_m_), effective PSII quantum yield (Φ_PSII_, (F_m_’ − F_t_)/F_m_), non-photochemical quenching (NPQ, F_m_/F_m_’ − 1) and electron transport rate (ETR), were determined. Here, F_v_ is the value of variable fluorescence, equal to the difference between Fm and F_0_; F_0_ is the minimum amplitude of fluorescence (F); and F_m_ is the maximum amplitude of fluorescence. F_m_ and F_m_’ are the maximum Chl fluorescence levels under dark- and light-adapted conditions, respectively. F_v_ is the photoinduced change in fluorescence, and F_t_ is the level of fluorescence before a saturation impulse is applied. F_0_ is the initial Chl fluorescence level. All parameters were calculated according to the methods reported by Pashkovskiy et al. (2021) [62].

### 5.6. Soluble Sugar, Sucrose and Starch Content

After 14 d of treatment, the fourth trifoliate leaf of alfalfa seedlings was collected for measurement of soluble sugar, sucrose and starch contents. Dried leaf tissues from all plants were ground to obtain homogenous samples, and subsamples were used to determine content of soluble sugar, sucrose and starch.

Soluble sugar and sucrose were extracted from the powdered sample (50 mg) three times, using 80% (*v*/*v*) ethanol at 80 °C. The supernatants were pooled and then diluted with 80% ethanol to 25 mL for the measurement of soluble sugar and sucrose content. Soluble sugar content was determined using the anthrone–sulfuric acid reagent method and calculated based on absorbance at a wavelength of 620 nm using the UV spectrophotometer [63]. Sucrose content was measured using the resorcinol method and estimated on the basis of the absorbance at a wavelength of 480 nm using the UV spectrophotometer [64]. The residue obtained after extraction was analyzed for starch, using the perchloric acid digestion method. Following extraction, starch content was determined photometrically in the presence of anthrone–sulfuric acid reagent and estimated on the basis of absorbance at a wavelength of 620 nm using the UV spectrophotometer [63].

### 5.7. Enzyme Activity

The second fully expanded leaf of alfalfa seedlings was harvested and used for enzymatic assays. The activity of enzymes, including ribulose-1,5-bisphosphate carboxylase/oxygenase (Rubisco, EC 4.1.1.39), ribulose-1,5-bisphosphate carboxylase/oxygenase activase (RCA, EC was not found), fructose-1, 6-bisphosphatase (FBPase, EC 3.1.3.11), thioredoxin reductase (TRXs, EC 1.8.1.9), sucrose synthase (SS, EC 2.4.1.13), sucrose phosphate synthase (SPS, EC 2.4.1.14), ADP-glucose pyrophosphorylase (AGPase, EC 2.7.7.27), soluble starch enzyme (SSE, EC 2.4.1.21), starch-branching enzyme (SBE, EC 2.4.1.18) and starch phosphorylase (SP, EC 2.4.1.1), was determined using plant-enzyme-linked immunosorbent assay (ELISA) kits. A frozen leaf sample (0.1 g) was homogenized in 1 mL of phosphate buffer (0.01 mol L^−1^, pH = 7.4) using a cold mortar and pestle and centrifuged at 5000× *g* and 4 °C for 10 min. The clear supernatant was then stored at 4 °C for 24 h pending analyses for the activity of enzymes. Firstly, 50 μL of standard or sample was added to the appropriate well of a microplate (except the blank wells). Secondly, 100 μL of HRP conjugate reagent was added, and the wells were covered with an adhesive plate membrane and incubated for 60 min at 37 °C. Thirdly, the liquid was discarded, and the wells were washed with 350 μL of wash buffer, and this procedure was repeated five times. Fourthly, a mixture of 50 μL of substrate A and 50 μL of substrate B was added to each well, mixed gently and incubated at 37 °C for 15 min in the dark. Finally, 50 μL of stop solution was added to each well, and the optical density was measured within 15 min at 450 nm using a microtiter plate reader (Infinite MPlex, Tecan, Austria). All the activity of enzymes was calculated using the methods reported by Pan et al. (2020) [18].

The protein concentration of each enzyme extraction solution was measured according to Li et al. (2020) [65]. The results are expressed as U/mL of protein.

### 5.8. Real-Time Quantitative PCR Verification

The second fully expanded leaf was harvested from five seedlings growing in each light treatment and used to determine RNA abundance. All the leaves were labeled and frozen in liquid nitrogen immediately. RNA was extracted using the TRIzol^TM^ Plus RNA Purification Kit (TaKaRa Biotechnology, Dalian, China). Reverse transcription and amplification of cDNA were performed using Super Script III First-Strand Synthesis Super Mix for qRT-PCR (Vazyme, Nanjing, China). Real-time quantitative PCR was conducted in Real-Time PCR System (CFX96, Bio-rad, USA), and 2^−∆∆CT^ method was used for data analysis [66]. The actin was selected as the reference gene. All target genes and target genes primers are listed in Supporting Information Table S1.

### 5.9. Statistical Analysis

All data analyses were conducted using one-way analysis of variance with the ANOVA packages of SPSS^®^ version 17.0 (SPSS Inc., Chicago, IL, USA). The homoscedasticity of the variables was determined using the Levene test. When the *F*-test indicated statistical significance was *p* < 0.05, Duncan’s new multiple range test for least significant difference (l.s.d.) was used to determine least significant range between means.

## Figures and Tables

**Table 1:** Effect of different light intensity treatments on specific leaf area (SLA, cm^2^ mg^−1^), shoot dry matter (SDM, mg plant^−1^), root dry matter (RDM, mg plant^−1^) and root-to-shoot ratio (RSR) of alfalfa plants.

Treatment ^a^	SLA	SDM	RDM	RSR
L100	0.474a (0.023)	205.4d (11.8)	22.0e (1.3)	0.108c (0.007)
L200	0.371b (0.021)	227.6d (17.7)	30.6d (2.2)	0.137b (0.013)
L300	0.269c (0.015)	320.4c (21.5)	54.2c (2.6)	0.170a (0.004)
L400	0.125d (0.004)	379.4b (10.6)	70.0b (3.2)	0.185a (0.007)
L500	0.117d (0.002)	461.4a (15.8)	82.2a (1.8)	0.179a (0.007)

^a^ L100, L200, L300, L400 and L500 refer 100, 200, 300, 400 and 500 µmol m^−2^ s^−1^, respectively. Within a column, values followed by different letters are significantly different (*p* <0.05). Values within parentheses are the standard errors of the means (*n* = 4).

**Table 2:** Effect of light intensity treatments on root length (RL, cm), root surface area (RSA, cm^2^), root volume (RV, cm^3^) and root diameter (RD, mm) of alfalfa plants.

Treatment ^a^	RL	RSA	RV	RD
L100	206.4e (7.2)	18.6e (0.7)	0.229e (0.011)	0.243c (0.013)
L200	397.1d (6.2)	44.4d (1.0)	0.787d (0.009)	0.325b (0.006)
L300	426.8c (8.7)	58.0c (2.2)	1.298c (0.081)	0.418a (0.012)
L400	474.6b (11.2)	67.0b (2.8)	1.650b (0.083)	0.430a (0.009)
L500	583.1a (5.7)	84.4a (2.7)	2.349a (0.179)	0.449a (0.010)

^a^ L100, L200, L300, L400 and L500 refer 100, 200, 300, 400 and 500 µmol m^−2^ s^−1^, respectively. Within a column, values followed by different letters are significantly different (*p* < 0.05). Values within parentheses are the standard errors of the means (*n* = 4).

**Table 3:** Effect of light treatments on Chlorophyll a (Chl a, μg cm^−2^), Chlorophyll b (Chl b, μg cm^−2^), carotenoids (Car, μg cm^−2^), Chl a + b (μg cm^−2^), Chl a/b and leaf nitrogen content (LNC, mg/g) of alfalfa plants.

Treatment ^a^	Chl a	Chl b	Car	Chl a + b	Chl a/b	LNC
L100	36.5a (1.7)	13.1a (0.3)	6.91a (0.24)	49.6a (1.8)	2.80b (0.14)	38.0a (1.1)
L200	34.1ab (2.0)	11.5ab (0.9)	6.19b (0.12)	45.6ab (2.9)	2.98ab (0.07)	28.0b (0.5)
L300	31.0bc (1.7)	10.3bc (0.8)	6.15b (0.21)	41.9bc (2.4)	3.10ab (0.12)	18.7c (0.2)
L400	29.7bc (1.1)	9.4c (0.4)	6.11b (0.13)	39.1c (1.4)	3.18a (0.12)	17.4c (0.6)
L500	28.6c (1.2)	8.7c (0.5)	5.49c (0.29)	37.3c (1.6)	3.29a (0.12)	18.8c (0.2)

^a^ L100, L200, L300, L400 and L500 refer 100, 200, 300, 400 and 500 µmol m^−2^ s^−1^, respectively. Within a column, values followed by different letters are significantly different (*p* < 0.05). Values within parentheses are the standard errors of the means (*n* = 4).

## Data Availability

The data presented in this study are available in the article and supplementary material.

## Supplementary Materials

The following supporting information can be downloaded at: https://www.mdpi.com/article/10.3390/plants11131688/s1. Figure S1. The spectral distribution of LED lights under different light intensity treatments. Table S1. List of primers for characterizing alfalfa genes (5′–3′) measured under different light treatments. *RCA*, *RbcL*, *RbcS*, *FBPase*, *TK* and *PGK* involve in Rubisco activase, Rubisco large subunit, Rubisco small subunit, Fructose-1,6-bisphosphatase, Transketolase, and Phosphoglycerate kinase, respectively. SS and SPS genes involve in sucrose synthase and sucrose phosphate synthase, respectively. *AGPase*, *SSS*, *SBE*, and *SP* involve in ADP-glucose pyrophosphorylase, soluble starch synthase, starch branching enzyme and starch phosphorylase, respectively. Table S2. Results of one-way ANOVA of effect of light intensity treatment on plant height, stem diameter, abaxial leaf petiole angle, specific leaf weight, shoot dry matter, root dry matter and root to shoot ratio of alfalfa plants. Table S3. Results of one-way ANOVA of effect of light intensity treatment on root length (RL), root surface (RS), root volume (RV), and root diameter (RD) of alfalfa plants. Table S4. Results of one-way ANOVA of effect of light intensity treatment on Chlorophyll a (Chl a), Chlorophyll b (Chl b), carotenoids (Car), Chl a + b, Chl a/b and leaf nitrogen content (LNC) of alfalfa plants. Table S5. Results of one-way ANOVA of effect of light intensity treatment on net photosynthetic rate (*P*_n_), transpiration rate (*T*_r_), intercellular CO_2_ concentration (*C*_i_), and stomatal conductance (*g*_s_) of alfalfa plants. Table S6. Results of one-way ANOVA of effect of light intensity treatment on maximal PSII quantum yield (F_v_/F_m_), effective PSII quantum yield (ΦPSII), non-photochemical quenching (NPQ) and electron transport rate (ETR) of alfalfa plants. Table S7. Results of one-way ANOVA of effect of light intensity treatment on soluble sugar (SS), sucrose, and starch (St) content of alfalfa plants.
