# Supplementary material for: Effect of Light Intensity on Morphology, Photosynthesis and Carbon Metabolism of Alfalfa (Medicago sativa) Seedlings
Source: Plants (Basel). 2022 Jun 25;11(13):1688. doi: 10.3390/plants11131688 (PMC9269066; doi:10.3390/plants11131688)
Supplement: Supplementary file 1 [file plants-11-01688-s001.zip › plants-1774453-supplementary.pdf]

Supplementary material

Effect of Light Intensity on Morphology, Photosynthesis and Carbon Metabolism of Alfalfa (*Medicago Sativa*) Seedlings

Number of Figures: 1

Number of Tables: 9

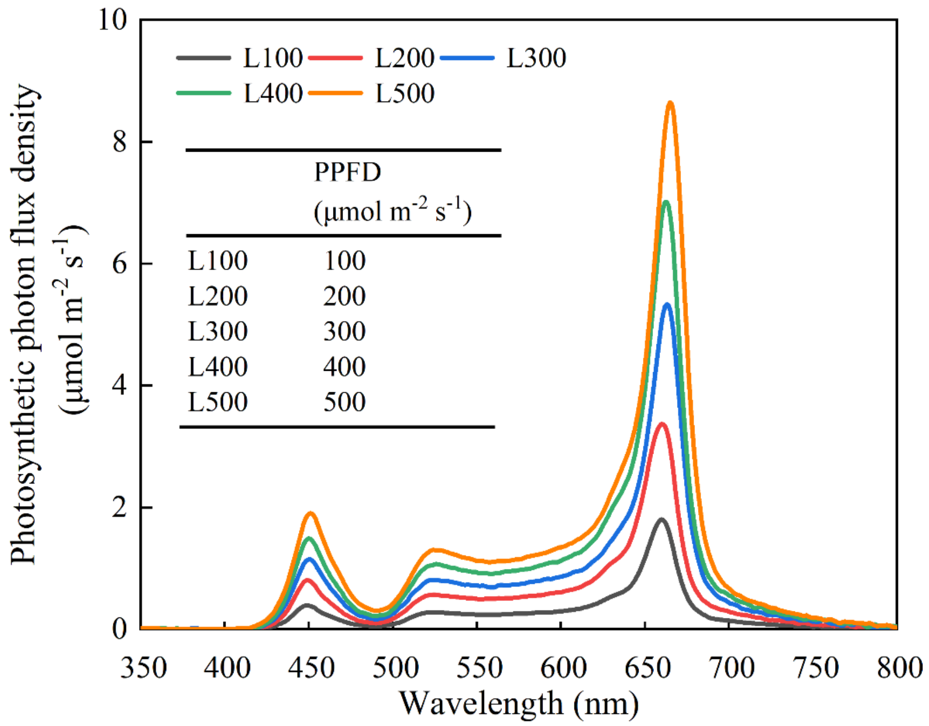

Figure S1. The spectral distribution of LED lights under different light intensity treatments.

Table S1. List of primers for characterizing alfalfa genes (5' - 3') measured under different light treatments. *RCA*, *RbcL*, *RbcS*, *FBPase*, *TK and PGK* involve in Rubisco activase, Rubisco large subunit, Rubisco small subunit, Fructose-1,6-bisphosphatase, Transketolase, and Phosphoglycerate kinase, respectively. *SS* and *SPS* genes involve in sucrose synthase and sucrose phosphate synthase, respectively. *AGPase*, *SSS*, *SBE*, and *SP* involve in ADP-glucose pyrophosphorylase, soluble starch synthase, starch branching enzyme and starch phosphorylase, respectively.

Location	Gene	Forward primer	Reverse primer
MS.gene63480.t1	*RCA*	CAGGTCGTATGGGTGGAACC	GAGTTGGACATTGGTGGGGT
FKU49_pgp074 *NC_042841.1*	*RbcL*	TTCCGAGTAAGTCCGCAACC	TCCATCGGTCCACACAGTTG
MS.gene54465.t1	*RbcS*	GAAGGGATGGGTTGCTTGCT	GGCAACTTCCACATTGTCCAG
MS.gene019792.t1	*FBPase*	GACCCTTCTCTTGGCGAGTT	ATCCCAGTTCTTGGCGTTCC
MS.gene74217.t1	*TK*	CACGCAATGGGAGCAATCTG	TGGCAGCTCTCATGTAGTCG
MS.gene92153.t1	*PGK*	CTTGGCACCTCTTGTTCCCA	CTGGAAGGGAAGCCACCAAC
MS.gene26705.t1	*SS*	GAGAGCGAGATGCTCAGTCG	GACTGCATCGGGGAGAAGAC
MS.gene036705.t1	*SPS*	TGACGTTGGTCCTGGTCTTG	GCCCGAACCCAAGAACGATA
MS.gene25721.t1	*AGPase*	ATTCTGTCGTTGGACTGCGA	GGAACACTGCCTTTAGCAGC
MS.gene06703.t1	*SSS*	AGGCGCTCAAGAAGTTGGTT	CCTTAAACGTCCCGTGCTTG
MS.gene051360.t1	*SBE*	AGTTAGGCTTCACACGCAGT	CCACACACCAAAATCGTCCC
MS.gene01471.t1	*SP*	AGTTTCCGACGAAGGTTGCT	AACCTCCCATGCTTCATCCC
	Actin	CCCCATTGAGCACGGTATTG	GGGTTAAGTGGAGCCTCAGT

Table S2. Results of one-way ANOVA of effect of light intensity treatment on plant height, stem diameter, abaxial leaf petiole angle, specific leaf weight, shoot dry matter, root dry matter and root to shoot ratio of alfalfa plants.

Source	df[a]	Plant height (cm)	Stem diameter (mm)	Abaxial leaf petiole angle (°)	Specific leaf area (cm^2 mg^{-1})	Shoot dry matter (mg $plant^{-1}$)	Root dry matter (mg $plant^{-1}$)	Root to shoot ratio
Treatment	4	22.5***	24.2***	44.0***	121.4***	44.2***	121.5***	16.4***

***; *F*-value significant at $P < 0.001$.

[a] df, degree of freedom

Table S3. Results of one-way ANOVA of effect of light intensity treatment on root length (RL), root surface (RS), root volume (RV), and root diameter (RD) of alfalfa plants.

Source	df[a]	RL (cm)	RS (cm^2)	RV (cm^3)	RD (mm)
Treatment	4	292.6***	144.2***	71.9***	73.1***

***; *F*-value significant at $P < 0.001$.

[a] df, degree of freedom

Table S4. Results of one-way ANOVA of effect of light intensity treatment on Chlorophyll a (Chl a), Chlorophyll b (Chl b), carotenoids (Car), Chl a + b, Chl a/b and leaf nitrogen content (LNC) of alfalfa plants.

Source	df[a]	Chl a (μg cm^{-2})	Chl b (μg cm^{-2})	Car (μg cm^{-2})	Chl a + b (μg cm^{-2})	Chl a/b	LNC (mg/g)
Treatment	4	4.1*	7.9***	5.8**	5.6**	2.6*	210.6***

*, **, and ***; *F*-value significant at $P < 0.05$, $P < 0.01$ and $P < 0.001$, respectively.

[a] df, degree of freedom

Table S5. Results of one-way ANOVA of effect of light intensity treatment on net photosynthetic rate (P_n), transpiration rate (T_r), intercellular CO_2 concentration (C_i), and stomatal conductance (g_s) of alfalfa plants.

Source	df[a]	P_n (μmol CO_2 m^{-2} s^{-1})	T_r (mmol H_2O m^{-2} s^{-1})	C_i (mol CO_2 mol^{-1})	g_s (mol H_2O m^{-2} s^{-1})
Treatment	4	73.9***	9.4***	26.9***	7.3**

, and *; F-value significant at $P < 0.01$ and $P < 0.001$, respectively.

[a] df, degree of freedom

Table S6. Results of one-way ANOVA of effect of light intensity treatment on maximal PSII quantum yield (F_v/F_m), effective PSII quantum yield (Φ_{PSII}), non-photochemical quenching (NPQ) and electron transport rate (ETR) of alfalfa plants.

Source	df [a]	F_v/F_m	Φ_{PSII}	NPQ	ETR
Treatment	4	16.4***	32.5***	20.8***	384.8***

***; *F*-value significant at $P < 0.001$.

[a] df, degree of freedom

Table S7. Results of one-way ANOVA of effect of light intensity treatment on soluble sugar (SS), sucrose, and starch (St) content of alfalfa plants.

Source	df[a]	SS (mg/g)	Sucrose (mg/g)	St (mg/g)
Treatment	4	185.9***	195.5***	481.1***

***; *F*-value significant at $P < 0.001$.

[a] df, degree of freedom

Table S8. Results of one-way ANOVA of effect of light intensity treatment on activity of Rubisco (Rubisco), ribulose-1,5-bisphosphate carboxylase/oxygenase activase (RAC), fructose-1,6-bisphosphatase (FBPase), and thioredoxin reductase (TRXs), sucrose synthase (SS) and sucrose phosphate synthase (SPS), adenosine diphosphate glucose pyro-phosphorylase (AGPase), soluble starch synthase (SSS), starch branching enzyme (SBE), and starch phosphorylase (SP) of alfalfa plants.

Source	df[a]	RAC ($U\ ml^{-1}$)	Rubisco ($U\ ml^{-1}$)	FBPase ($U\ ml^{-1}$)	TRXs ($U\ ml^{-1}$)	SS ($U\ ml^{-1}$)	SPS ($U\ ml^{-1}$)	AGPase ($U\ ml^{-1}$)	SSS ($U\ ml^{-1}$)	SBE ($U\ ml^{-1}$)	SP ($U\ ml^{-1}$)
Treatment	4	156.4***	472.7***	48.6***	361.3***	25.1***	44.5***	110.2***	250.4***	86.8***	1105.4***

***; *F*-value significant at $P < 0.001$.

[a] df, degree of freedom

Table S9. Results of one-way ANOVA of effect of light intensity treatment on gene expression level of *RCA*, *RbcL*, *RbcS*, *FBPase*, *TK*, *PGK*, *SPS*, *SS*, *AGP*, *SSS*, *SBE*, and *SP* of alfalfa plants.

Source	df[a]	*RCA*	*RbcL*	*RbcS*	*FBPase*	*TK*	*PGK*	*SPS*	*SS*	*AGP*	*SSS*	*SBE*	*SP*
Treatment	4	313.6***	194.4***	121.2***	52.4***	110.1***	80.4***	126.8***	269.3***	368.9***	341.9***	135.2***	400.9***

***; *F*-value significant at $P < 0.001$.

[a] df, degree of freedom
